# Prevalence of pneumonia and its determinant factors among under-five children in Gamo Zone, southern Ethiopia, 2021

**DOI:** 10.3389/fped.2022.1017386

**Published:** 2022-12-22

**Authors:** Yerukneh Solomon, Zelalem Kofole, Tewodros Fantaye, Solomon Ejigu

**Affiliations:** ^1^ Department of Biomedical Sciences, College of Medicine and Health Sciences, Debre Berhan University, Debre Berhan, Ethiopia; ^2^ Department of Biomedical Sciences, School of Medicine, College of Health Science, Arba Minch University, Arba Minch, Ethiopia; ^3^ Department of Biomedical Sciences, College of Medicine and Health Science, Mizan-Tepi University, Mizan-Aman, Ethiopia

**Keywords:** pneumonia, childhood illness, Arba Minch, Gamo, under-five

## Abstract

**Background:**

Pneumonia, which is a form of acute lower respiratory tract infection, affects the lung parenchyma and destructs alveolar air space. Pneumonia is the leading cause of morbidity and mortality in under-five children. It was estimated that pneumonia kills 900,000 under-five children each year worldwide. Approximately 172 deaths per 1,000 live births occur in sub-Saharan African countries, with pneumonia being the major cause. This study aimed to assess the prevalence and determinant factors of pneumonia inunder-five children in southern Ethiopia.

**Methodology:**

An institutional cross-sectional study was employed. A total of 239 child–caregiver pairs were included. Data were collected by trained nurses using a semi-structured questionnaire. The collected data were checked for completeness, coded and entered into EPI data version 4.6, and exported to SPSS version 25 for analysis. Results were reported as the mean, frequency, and percentile. Logistic regression was employed to assess statistically significant predictors of pneumonia. Variables with a *p*-value <0.05 were considered statistically significant factors of pneumonia.

**Result:**

The prevalence of pneumonia in the study area was 30%. Among the factors assessed, place of food cooking—inside the living room [adjusted odd ratio (AOR) = 5.79, 95% confidence interval (CI): 2.47–13.58], nonexclusive breastfeeding (AOR = 3.26, 95% CI: 1.42–7.52), vitamin A supplementation status (AOR = 5.62, 95% CI: 2.65–11.94), and vaccination status (AOR = 3.59, 95% CI: 1.49–8.66) were significantly associated with the occurrence of pneumonia in under-five children.

**Conclusion:**

This study showed that the prevalence of pneumonia was relatively higher in Arba Minch town than other parts of the country. Place of food cooking, nonexclusive breastfeeding, vitamin A supplementation status, and vaccination status of children were significant factors of pneumonia among under-five children. Enhancing caregivers’/mothers’ awareness of predicted factors was needed to reduce the incidence of childhood pneumonia and to enhance children's quality of health.

## Introduction

Pneumonia is a acute lower respiratory tract infection that affects the lung parenchyma and destructs alveolar air space (1). Pneumonia is the leading cause of morbidity and mortality in under-five children. In 2016, it was estimated that 900,000 under-five children died of pneumonia worldwide (2). From these, sub-Saharan African countries took the lion's share with 50% of the burden of worldwide under-five children mortality rate due to pneumonia. Approximately 172 deaths per 1,000 live births occur in sub-Saharan African countries, mainly due to pneumonia (3, 4). Between 2000 and 2019, the global under-five children mortality rate decreased by about 50%, but progress is still slower, and 65 (32%) of 204 countries, primarily in sub-Saharan Africa and south Asia, are not on course to fulfil either sustainable development goal (SDG) 3.2 targeted by 2030 (5). Nearly half of all under-five deaths in 2019 occurred in just five countries: Nigeria, India, Pakistan, the Democratic Republic of Congo and Ethiopia. Globally, infectious diseases, including pneumonia, diarrhea, and malaria, remain a leading cause of under-five deaths (6). Children with compromised immune systems like malnutrition, especially those not exclusively fed under-five children, are at a higher risk of developing pneumonia (7).

In developing countries like Ethiopia, the risk factors for pneumonia in under-five children are numerous. Among factors, nonexclusive breastfeeding, lack of/incomplete immunization, environmental conditions, outdoor/indoor air pollution, micronutrients, and vitamin deficiencies are predominantly reported (8–10).

The World Health Organization's Integrated Management of Childhood Illness (IMNCI) recommendations have been used in Ethiopia, which has done so since 2001 (11). Additionally, Ethiopia made a policy breakthrough by introducing community-based treatment of pneumonia through health extension workers in 2010. This was done to increase access to lifesaving interventions. Since then, integrated community case management has been used to treat pneumonia in the community by health extension workers over the whole health posts in the country. Even though child mortality due to pneumonia decreased, progress among under-five children is still slow (12).

In Ethiopia, pneumonia is one the leading causes of death in under-five children, with an estimated contribution of more than 40,000 deaths annually (13, 14). These deaths could be prevented by cost-effective interventions like immunization, health education, good nutrition, exclusive breastfeeding, and sanitary management (9). Reports showed that the prevalence of pneumonia among under-five children in Ethiopia reached 20.68% (14). According to a study conducted in Wondo Genet, southern Ethiopia, the magnitude of pneumonia in under-five children visiting health centers was reported to be 33.5% (15). Globally, Ethiopia ranks sixth among the top 15 countries in morbidity and mortality from pneumonia (16). Predictive factors of pneumonia need to be studied to better inform healthcare providers and other stakeholders/policymakers to consider additional pneumonia prevalence reduction strategies. Although studies are conducted in Ethiopia, the determinant factors for pneumonia differ in different societies and study populations (17). Taking the high burden of pneumonia and the variability of risk factors into account, this study aimed to identify the predicted factors of pneumonia among under-five children. Furthermore, there is no previous study in the area that could show the current status of pneumonia and its determinant factors.

## Method and materials

### Source population

All under-five children visiting the Pediatrics Outpatient Department of Arba Minch General Hospital.

### Study population

All under-five children visiting the Pediatrics Outpatient Department of Arba Minch General Hospital during the data collection period.

### Inclusion and exclusion criteria

The study included children who were under 5 years of age, those who were residents of Arba Minch town for a minimum of 6 months, and those who visited the pediatric unit of Arba Minch referral Hospital during the study period. Children with the following conditions were excluded from the study: cough that lasted for more than 15 days, cough because of the recent history of aspiration of liquid or foreign body, cardiac disease, and caregiver who did not have any information about the child during the time of data collection.

### Sample size determination

The study utilized the single population proportion formula technique to determine the study population sample size

$$n=\frac{\left[{\left(Z\alpha /2\right)}^{2\;}\times P\left(1-P\right)\right]}{d^{2}}$$

where *n* is the desired sample size,

*P* is the population of under-five pneumonia children, which is 28.1% (18), have taken from previously published study from jimma town
*Z_α_*_/2_ is the critical value at the 95% confidence interval level of certainty (1.96), and
*d* is the margin of error between the sample and population (5%)

$$n=\frac{\left[{\left(1.96\right)}^{2}\times 0.281\left(1-0.281\right)\right]}{{\left(0.05\right)}^{2}}=\frac{0.776}{0.0025}=310.4\approx 310$$

where source population (*N*)= 728, which was a monthly plan on the proportion of under-five children visiting the outpatient department in Arba Minch General Hospital. Because the source population was less than 10,000, we can use the correction formula to get the actual sample size of the study population: $nf=\frac{n}{1+n/N}$

where

*nf* = final sample size,
*n* = first calculated sample size,
*N* = source population.

$nf=\frac{310}{(1+\frac{310}{728})\;\;}=217$. By adding 10% of the nonresponse rate, the total sample size of this study was 217 + 22 = 239.

### Sampling procedure

A total of 239 participants were enrolled in this study. The study participants were selected by using the simple random sampling technique from under-five children outpatient registration book of Arba Minch General Hospital. The first study participant was selected by using *K*-value. That is

$$K=N/nf=\frac{\begin{array}{c} \mathrm{Total}\;\mathrm{under}\;\mathrm{five}\;\mathrm{children}\;\mathrm{visiting}\;\mathrm{OPD}\\ \;\mathrm{of}\;\mathrm{the}\;\mathrm{hospital}\;\mathrm{during}\;\mathrm{data}\;\mathrm{collection} \end{array}}{\;\mathrm{final}\;\mathrm{sample}\;\mathrm{size}}=\frac{728}{239\;}=3$$

The first was selected from 1 − *k* (that is, from 1 to 3) by using the lottery method on the first day of data collection.

### Study variables

Dependent variables: Pneumonia among under-five children.

Independent variables:

Sociodemographic factors: Age, sex, and residence.

Health facility and childcare factors: Low birth weight, prematurity, exclusive breastfeeding, breastfeeding duration, vaccination status, and vitamin “A” supplementation.

### Environmental risk factors

Cook food inside the living room, home aeration, hand washing practice, domestic smoking, and carrying baby during cooking.

### Data collection procedures and tools

The data collection tool was developed after reviewing previously published relevant literature (14, 18, 19). The adopted structured questionnaire included sociodemographic factors, environmental factors, and health facility and childcare factors. Before data collection was started, the questionnaire was pretested on a 5% sample size at Chencha Primary Hospital to ensure the validity and reliability of the study tool. After collecting the pretest data, it was checked for potential problems related to the tool, such as any difficult question that was not easily understandable and unclear to reply, and corrective measures were taken. The data were collected through face-to-face interviews and medical chart reviews. Charts were reviewed to collect information about the diagnosis, pre-existing or comorbidities, and anthropometric data. Data were collected by four diploma nurses and one first-degree nurse who received 2 days of training on the objective and contents of the study.

### Data quality assurance and analysis

The collected data were checked for completeness, coded and entered into Epi Info version 6, and exported to SPSS version 25 for analysis. Then, data were cleaned for consistency and the extent of outliers. Different statistical assumptions and appropriate corrections were made prior to analysis. Descriptive analyses were made for each independent variable. Bivariate and multivariate binary logistic regression analyses were used to test the association between the independent and dependent variables. Bivariate analysis was performed for each of the independent variables with the outcome variable. Variables with a *p*-value of <0.25 on bivariate analysis were taken as candidates for multivariate binary logistic regression model analysis to identify predictors of the outcome variable. Variables with a *p*-value of <0.05 on multivariate binary logistic regression analysis were considered predictive factors for pneumonia among under-five children. The strength of the association between the outcome variable and independent variables was expressed using an adjusted odd ratio (AOR) with 95% confidence intervals (CIs).

## Results

### Sociodemographic characteristics of study participants

A total of 239 children's mothers/caregivers have participated in the current study, which made a response rate of 100%. About 125 (52.3%) of the children were boys. The highest proportions (41%) of participating children were in the age group of 12–36 months. In the current study, 75% of the respondents (children's mothers/caregivers) had formal education. Regarding the marital status of children's mothers/caregivers, 230 (96.2%) of the participants were married. The occupational profile of children's mothers/caregivers showed that the majority (189, 79%) were self-employed and 30 (12.5%) were civil servants. More than half (54.8%) of the study participants were residing in rural parts of the study area (shown in Table 1).

**Table 1:** Sociodemographic characteristics of children and mothers/caregivers studied at the Arba Minch General Hospital, southern Ethiopia (2021).

Variables	Category	Frequency	Percent
Sex	Male	125	52.3
	Female	114	47.7
Age of child (months)	2–12	71	29.7
	13–36	98	41
	37–59	70	29.3
Residence of child	Urban	131	54.8
	Rural	108	45.2
Family’s monthly income Ethiopian Birr	<2,000	31	13
	2,001–3,999	122	51
	4,000–5,999	62	25.9
	≥6,000	24	10
Mother’s marital status	Married	230	96.2
	Unmarried	9	3.8
Mother’s educational status	Illiterate	58	24.3
	Literate	181	75.7
Mother’s occupation	Self-employed	189	79.1
	Unemployed	11	4.6
	Civil servant	30	12.5
	Other	9	3.7

### Prevalence of pneumonia and its signs and symptoms among participants

**Figure 1:**
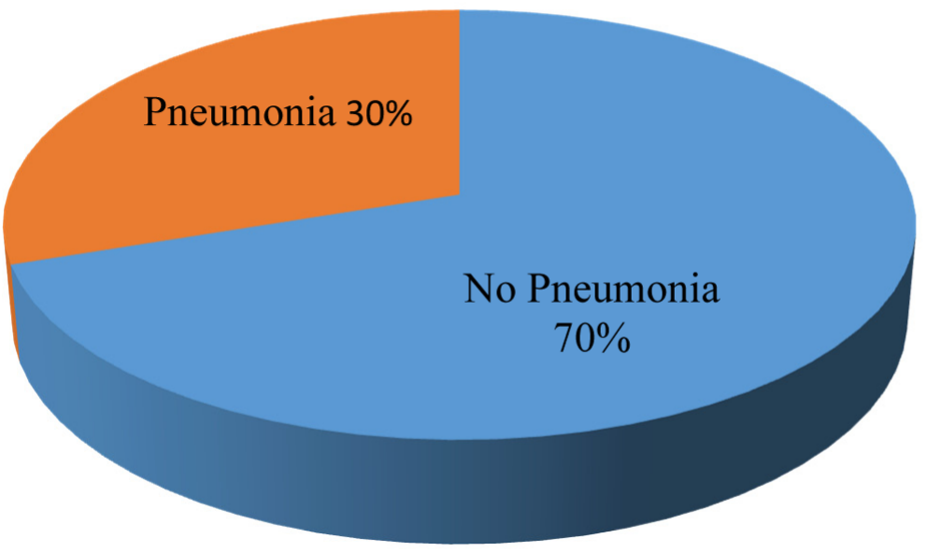
Pneumonia among under-five children visiting the Arba Minch General Hospital in 2021.

**Figure 2:**
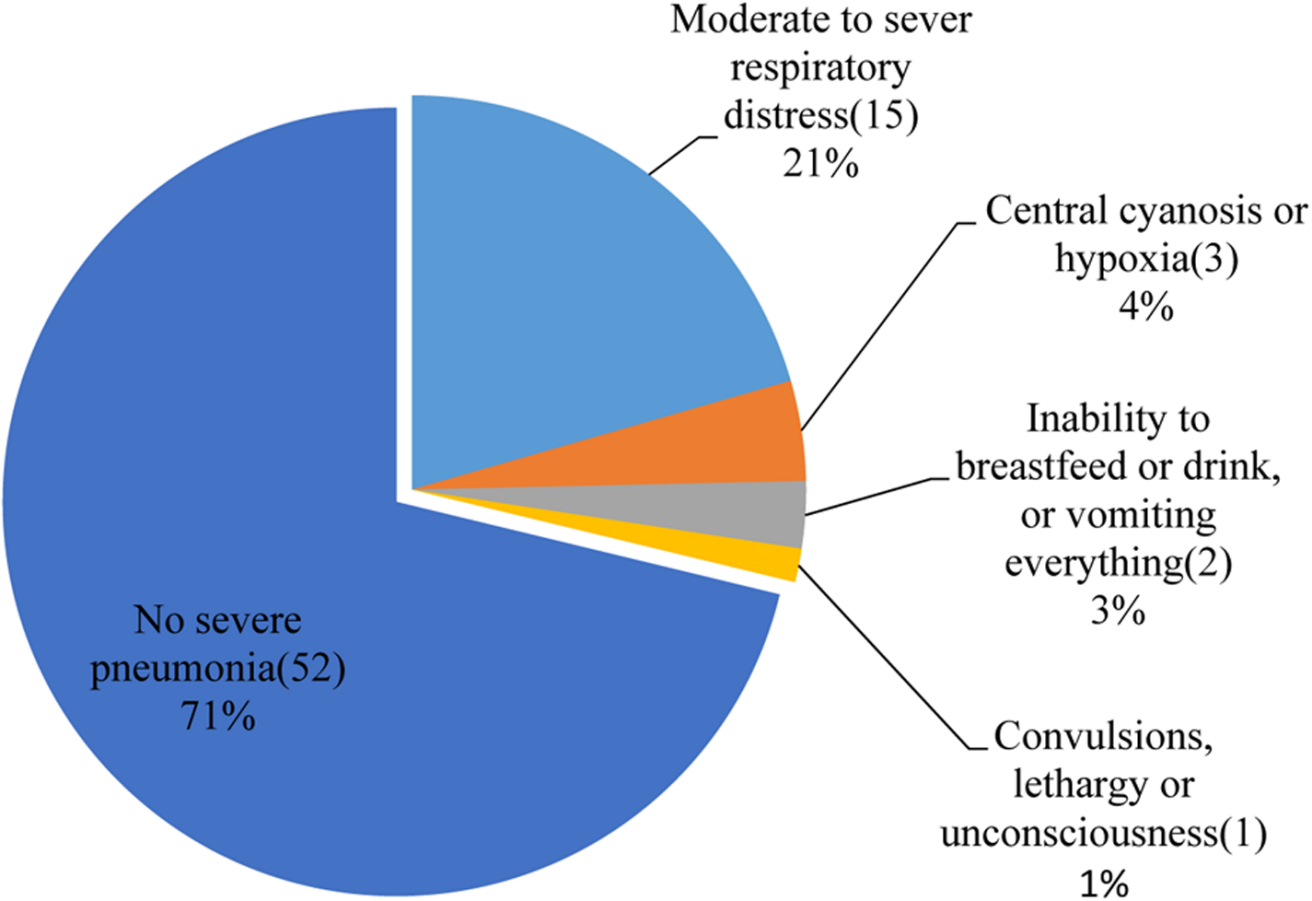
Severe pneumonia among under-five children visiting the Arba Minch General Hospital in 2021.

From the total participating children, 38% had a history of cough, 18% had difficulty breathing, and 34% had fast breathing at the time of the study. In addition to these signs and symptoms, 169 (70.7%) had a fever, and 35 (14.6%) had chest in-drawing. Our study showed that the overall prevalence of pneumonia among under-five children during the study period in Arba Minch General Hospital was 30% (73) (as shown in Figure 1). Among the children diagnosed with pneumonia, 21 (28.8%) had severe pneumonia and 15 (71.4%) had moderate to severe respiratory distress (as shown in Figure 2).

### Factors affecting the occurrence of pneumonia among under-five children

Bivariate analysis showed that cooking food inside the living room, nonexclusive breastfeeding in the first 6 months, vitamin A supplementation status, caring for child on mothers’/caregivers’ back or beside the mother during food cooking, kitchen without a window, and children's contact history of a family with acute respiratory tract infection were significantly associated with pneumonia among under-five children (shown in Table 2). Variables with a *p*-value of <0.25 in the bivariate analysis were candidates for multivariate analysis. Multivariate analysis showed that the kitchen not being separated from the main house for food cooking, nonexclusive breastfeeding, vitamin A supplementation status, and vaccination status of children were statistically significant factors to be considered as independent causes of pneumonia in under-five children (shown in Table 3).

**Table 2:** Bivariate logistic regression analysis of independent variables and occurrence of pneumonia in under-five children in the Arba Minch General Hospital, 2021.

Independent variable	Category	Pneumonia		COR (95% CI)	*p*-value
		Yes	No		
Place of cooking	Living room	64	96	6.757 (3.329–13.715)	0.001
	Kitchen	9	70	1	
Breastfeeding status	Nonexclusive	47	70	2.922 (1.527–5.592)	0.001
	Exclusive	26	96	1	
Vitamin A supplementation	No	64	86	5.621 (2.645–11.94)	0.001
	Yes	19	70	1	
Contact history of the family with acute RTI	Yes	24	96	2.80 (1.752–4.988)	0.002
	No	49	70	1	
Vaccination status	Fully	37	114	1	
	Up to date	14	20	3.000 (1.994–9.051)	0.051
	Partial	7	14	1.146 (0.559–2.350)	0.710
	Unvaccinated	15	18	2.270 (1.265–4.075)	0.006
Location of the child while cooking	On mother back	25	61	1.901 (1.992–3.644)	0.053
	Outside cooking room	70	83	1	
Kitchen with a window	Yes	24	96	1.812 (1.883–3.721)	0.105
	No	49	70	1	

CI, confidence interval; COR, crude odd ration; RTI, respiratory tract infection.

**Table 3:** Logistic regression analysis of independent variables and occurrence of pneumonia in under-five children in the Arba Minch General Hospital, 2021.

Independent variable	Category	Child diagnosis of pneumonia		AOR (95% CI)	*p*-value
		Yes	No		
Place of cooking	Living room	64	96	5.791 (2.469–13.584)	0.001
	Kitchen	9	70	1	
Breastfeeding status	Mixed	47	70	3.262 (1.415–7.522)	0.002
	Exclusive	26	96	1	
Vitamin A supplementation	No	64	86	4.803 (2.260–10.205)	0.001
	Yes	19	70	1	
Vaccination status	Fully	37	114	1	
	Up to date	14	20	1.416 (0.659–3.043)	0.372
	Partial	7	14	1.046 (0.679–2.420)	0.610
	Unvaccinated	15	18	3.59 (1.491–8.662)	0.004

AOR, adjusted odds ratio; CI, confidence interval.

## Discussion

In this study, the kitchen not being separated from the main house for food cooking, nonexclusive breastfeeding, vitamin A supplementation status, and vaccination status of children were found to be significant risk factors associated with the occurrence of pneumonia among under-five children. This study also showed a high prevalence of pneumonia, with 30% among under-five children (as shown in Figure 1). Our finding was in agreement with a study done in other parts of Ethiopia (15, 18). However, the result of this study was greater than that of the study conducted by Merkeb and Fentahun(20.68%) (14) and lower than that of a study conducted in Uganda (53.7%) (20). This difference may be due to the study design (Merkeb and Fentahun conducted a systematic review and meta-analysis) and socioeconomic status variations and health service facility accessibility in the study area for the study in Uganda. Although the magnitude of pneumonia is reduced from time to time due to different health policies and strategies, pneumonia remains the leading cause of death in under-five children in sub-Saharan and developing countries. Utilization of effective strategies for the sustainable reduction of pneumonia in under-five children was hindered by many different factors (4). Our current study showed that under-five children from the household who used the main house for a place of cooking were 5.8 times (AOR = 5.791, 95% CI: 2.469–13.584, *p* = 0.001) more likely to develop pneumonia than those from the family having a separated kitchen (as shown in Table 3). Our study result was in line with other studies conducted in Wondo Genet, southern Ethiopia (15), a study conducted in Estie town Northwest Ethiopia (21), a study by Markos et al. (19), and a study conducted in central India and Dominican Republic (22, 23). The association between pneumonia and cooking environment might be due to the use of wood as a fuel produces irritants in the form of smoke. Inhalation of this smoke results in the impairment of alveolar cells and macrophages of the lung. This study indicated that unvaccinated under-five children were 3.5 times [AOR = 3.59, 95% CI: 1.491–8.662, *p* = 0.004] more vulnerable to suffering from pneumonia as compared to those children who completed their vaccination (as shown in Table 3). This result was supported by different studies conducted on predictors of pneumonia (14, 18, 24). This association could be due to the loss of strong enough immunity for causative agents. Concerning breastfeeding in the first 6 months of the children's age, the odds of being vulnerable to developing pneumonia was 3.3 times (AOR = 3.262, 95% CI: 1.415, 7.522, *P* = 0.002) more likely among children who were not exclusively breastfed as compared to those under-five children who were exclusively breastfed (Table 3). The finding was supported by Ramezani et al.’s report on the predictors of pneumonia (10) and was nearly similar to the UNICEF 2012 report on pneumonia predictors (24). This study also found that under-five children who were not supplemented by vitamin A were nearly five times (AOR = 4.803, 95% CI: 2.260–10.205, *p* = 0.001) more likely to suffer from pneumonia as compared to children who had vitamin A supplementation (Table 3). Studies in Rwanda also reported similar results (25). This association could be explained by the fact that vitamin A is an essential micronutrient that governs many biological processes and a deficiency of vitamin A will cause an imbalance between pro- and anti-inflammatory factors and excessive immune response (26).

### Limitations of the study

Because this study was institution-based research with data gathered from a single hospital in Ethiopia, conclusions may not be easily extrapolated to patients admitted to other areas. Second, even though the cases of pneumonia were classified by physicians using 2014 WHO standard clinical and integrated management of newborn child illness, this study did not use chest x-ray, blood cultures, and other cultures to confirm pneumonia. We merely used physician's diagnosis from patient cards. Hence, this may not be as reliable as pneumonia confirmation using laboratory diagnostic methods.

## Conclusion

This study showed that the prevalence of pneumonia was higher in Arba Minch town compared to study reports from other parts of the country. Place of food cooking, nonexclusive breastfeeding, vitamin A supplementation status, and vaccination status of children were significant factors of pneumonia among under-five children. Enhancing caregivers’/mothers’ awareness of predicted factors needed to reduce the incidence of childhood pneumonia and to increase enhanced children's quality of health.

## Data Availability

The original contributions presented in the study are included in the article/Supplementary Material; further inquiries can be directed to the corresponding author.
